# An elective radiation dose of 46 Gy is feasible in nasopharyngeal carcinoma treated by intensity-modulated radiotherapy: A long-term follow-up result

**DOI:** 10.1097/MD.0000000000006036

**Published:** 2017-02-10

**Authors:** Tsung-Min Hung, Kang-Hsing Fan, Eric Yen-Chao Chen, Chien-Yu Lin, Chung-Jan Kang, Shiang-Fu Huang, Chun-Ta Liao, Shu-Hang Ng, Hung-Ming Wang, Joseph Tung-Chieh Chang

**Affiliations:** a Department of Radiation Oncology, Chang Gung Memorial Hospital and Chang Gung University, Taoyuan; b Department of Radiation Oncology, Chang Gung Memorial Hospital, Keelung; c Department of Otorhinolaryngology, Head and Neck Surgery,; d Department of Diagnostic Radiology; e Division of Hematology/Oncology, Department of Internal Medicine, Chang Gung Memorial Hospital and Chang Gung University, Taoyuan, Taiwan.

**Keywords:** elective irradiation, intensity-modulated radiotherapy, nasopharyngeal carcinoma, radiation dose, treatment outcome

## Abstract

The purpose of this study is to compare the treatment outcome of different radiation doses of elective neck irradiation (ENI) in nasopharyngeal carcinoma (NPC) patients treated with intensity-modulated radiotherapy (IMRT).

In total, 504 patients with nondisseminated NPC who underwent magnetic resonance imaging before radical IMRT between 2000 and 2008 were retrospectively reviewed. The patients were classified into 2 groups based on the ENI dose: low ENI when the ENI dose was 46 Gy (n = 446) and high ENI when the ENI doses were 50 to 60 Gy (n = 58). All the patients in both the groups received a median dose of 72 Gy to the gross tumor and involved nodes. The fraction size was 2 Gy per fraction. Matching was performed between low ENI and high ENI in a 2:1 ratio, and the matching criteria were N-stage, T-stage, treatment modality, pathology classification, sex, and age.

The median follow-up for all patients was 63.5 months. In all patients, the 5-year progression-free survival (PFS), local control (LC), regional control (RC), distant metastasis-free survival (DMFS), overall survival (OS), and cancer-specific survival (CSS) for low ENI and high ENI patients were 69.0% and 63.2% (*P* = 0.331), 89.0% and 83.9% (*P* = 0.235), 90.1% and 85.2% (*P* = 0.246), 86.8% and 76.6% (*P* = 0.056), 77.5% and 80.8% (*P* = 0.926), and 84.4% and 82.5% (*P* = 0.237), respectively. In the matched-pair analysis, the 5-year PFS, LC, RC, DMFS, OS, and CSS for matched low ENI and high ENI patients were 74.1% and 63.2% (*P* = 0.134), 92.0% and 83.9% (*P* = 0.152), 90.1% and 85.2% (*P* = 0.356), 86.2% and 76.6% (*P* = 0.125), 87.0% and 80.8% (*P* = 0.102), and 88.6% and 82.5% (*P* = 0.080), respectively. In the multivariable analysis for all patients, the ENI group was not a significant factor for PFS, LC, RC, DMFS, OS, and CSS.

A low ENI dose of 46 Gy in 23 fractions is feasible in NPC patients treated with IMRT, and this concept should be validated in the prospective studies.

## 1 Introduction

Nasopharyngeal carcinoma (NPC) has a high incidence of lymph node metastasis due to the rich submucosal lymphatic network of the nasopharynx. Elective neck irradiation (ENI) covering the nodal regions with potential microscopic disease is the standard practice for radiation therapy (RT) for NPC.

The radiation dose of ENI for high-risk subclinical disease is 59.4 Gy in 33 fractions, according to Radiation Therapy Oncology Group studies of NPC.^[1,2]^ However, the elective radiation dose for prostate cancer, which is thought to be relatively radioresistant, is 46 to 50.4 Gy in conventional fractionation.^[3,4]^ In addition, the textbook of radiotherapy written by Gilbert H. Fletcher stated that 45 Gy in conventional fractionation is adequate for treating the microscopic disease of NPC (Table 3-39, page 380).^[5]^

In our hospital, the ENI dose of NPC ranges from 46 to 60 Gy and is determined at the discretion of the treating radiation oncologist. This study was undertaken to evaluate the treatment outcome of different ENI doses and to determine if an ENI dose as low as 46 Gy in 23 fractions is adequate to eradicate the microscopic disease of NPC.

## 2 Materials and methods

### 2.1 Patient selection and treatment

This study was approved by the Institutional Review Board of our hospital. In total, 504 patients with biopsy-proven, nondisseminated, newly diagnosed NPC who underwent magnetic resonance imaging (MRI) examination before radical intensity-modulated radiotherapy (IMRT) between November 2000 and December 2008 were retrospectively reviewed. The pretreatment evaluation included a head and neck MRI, a nasopharyngeal fiberoscopy, a bone scan, a chest X-ray, and an abdominal sonogram. The bone scan was optional when [^18^F]-fluorodeoxyglucose positron emission tomography was performed. A total of 437 (86.7%) patients underwent a positron emission tomography (PET) study before treatment; the details of the PET study have been previously published.^[6,7]^ All patients were restaged according to the seventh edition of the American Joint Committee on Cancer (7^th^ AJCC) staging system. The primary treatment consisted of RT alone for stage I disease and cisplatin-based concurrent chemoradiotherapy (CCRT) for stage II-IVb disease. Patients with advanced disease did not receive CCRT when medical comorbidities or patient refusal occurred. Thirty-one stage IVA-B patients participating in a randomized trial received epirubicin-based induction chemotherapy before CCRT. The median radiation dose to the gross tumor and nodes was 72 Gy (range, 70–76 Gy), and IMRT was used in all patients.

### 2.2 Elective neck irradiation groups

The patients were divided into 2 groups according to the ENI radiation dose, low ENI, or high ENI, at the discretion of the treating radiation oncologist. In the low ENI group, the radiation dose to all subclinical diseases was 46 Gy in 23 fractions. In the high ENI group, the radiation dose was 60 Gy in 30 fractions to the high-risk subclinical disease (first-echelon nodes) and 50 Gy in 25 fractions to the low-risk subclinical disease (regional nodal area except gross and first-echelon nodes). In the 446 patients of the low ENI group, the Ib region (the submandibular nodes) was not included in the elective radiation field because our previous study indicated that the involvement of the submandibular nodes is rare.^[8]^ The patient characteristics of the 2 ENI groups are summarized in Table 1. The median age of all patients was 48.6 years old (range 15–84 y/o), so we chose age 50 to group the patients. Both the low and high ENI groups had a median radiation dose of 72 Gy (range, 70–76 Gy) to the gross tumor and involved nodes. The staging PET study was performed in 87.7% of low ENI and 79.3% of high ENI group patients. To validate the treatment outcomes between the different ENI groups, we performed a 2:1 ratio match between the low and high ENI patients for matched-pair analysis (Table 2). The matching criteria were, in order, N-stage, T-stage, treatment modality, pathology classification, sex, and age.

**Table 1:**
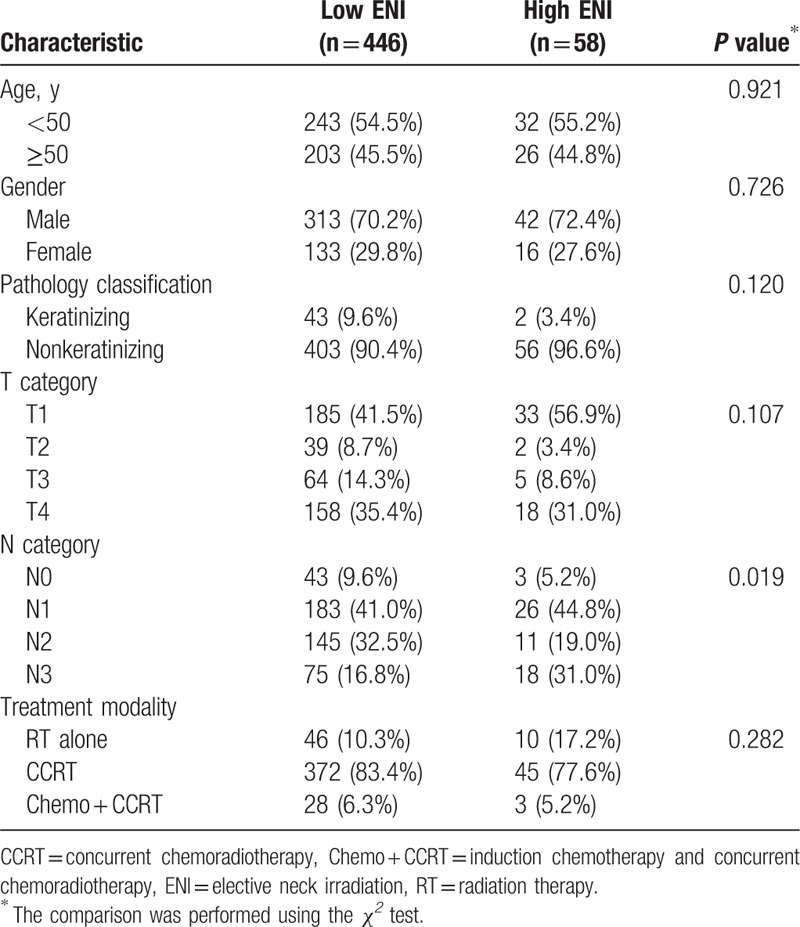
Patient characteristics of the ENI groups in all patients (n = 504).

**Table 2:**
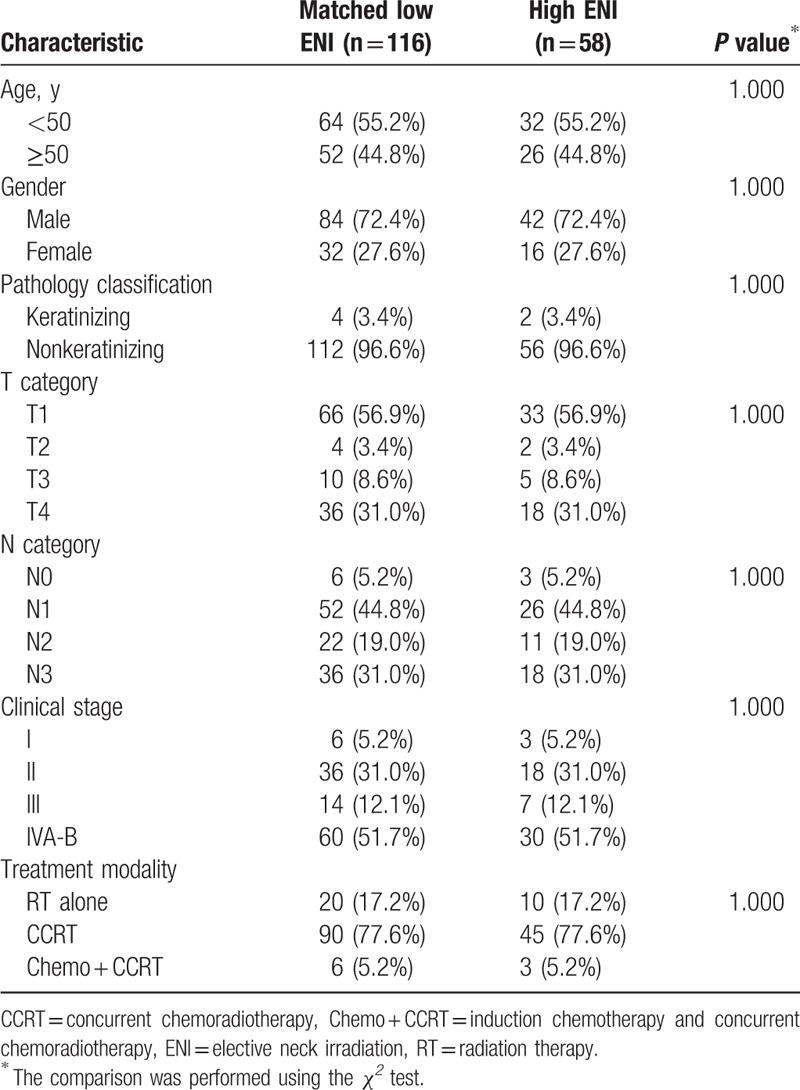
Patient characteristics in the matched-pair groups (n = 174).

### 2.3 Follow-up

The details regarding the follow-up plan for all patients have been previously published.^[9]^ In brief, all patients received MRI examination with or without PET study at 3 months after treatment, and a follow-up imaging study was performed when there was suspicion of disease recurrence or at least yearly in patients without clinical symptoms or signs. Disease recurrence was confirmed by biopsy to obtain tissues for histological confirmation if possible, and close clinical and image follow-up was pursued if a biopsy was not feasible or yielded a negative result. Complete restaging examinations, including PET, were highly recommended for patients with disease recurrence. Salvage treatment (RT, surgery, or chemotherapy) was provided for relapsed patients, and the treatment type was based on patient preference and performance status.

### 2.4 Statistical analysis

The following endpoints were evaluated: progression-free survival (PFS), local control (LC), regional control (RC), distant metastasis-free survival (DMFS), overall survival (OS), and cancer-specific survival (CSS). The failure of PFS was local, regional, or distant progression or death as a result of any cause. Survival was defined as the time between the date of RT initiation and the date of failure or last follow-up. The survival function was estimated using the Kaplan–Meier method. Univariate analysis was performed using the log-rank test. The *χ*^*2*^ test was used to compare the clinical features of the ENI groups. In multivariable analysis, the Cox proportional hazard model was used to test the independent significance. The following variables were included in the multivariable analysis: age (<50 years vs ≥50 years), gender (female vs male), pathology (keratinizing vs nonkeratinizing), T category (T1 vs T2 vs T3 vs T4), N category (N0 vs N1 vs N2 vs N3), chemotherapy (none vs concurrent vs induction + concurrent), and the ENI group (low vs high). A *P* value of <0.05 was considered statistically significant. All statistical analyses were performed using SPSS (Statistical Package for the Social Sciences) 13.0 software (SPSS Inc, Chicago, IL).

## 3 Results

**Figure 1:**
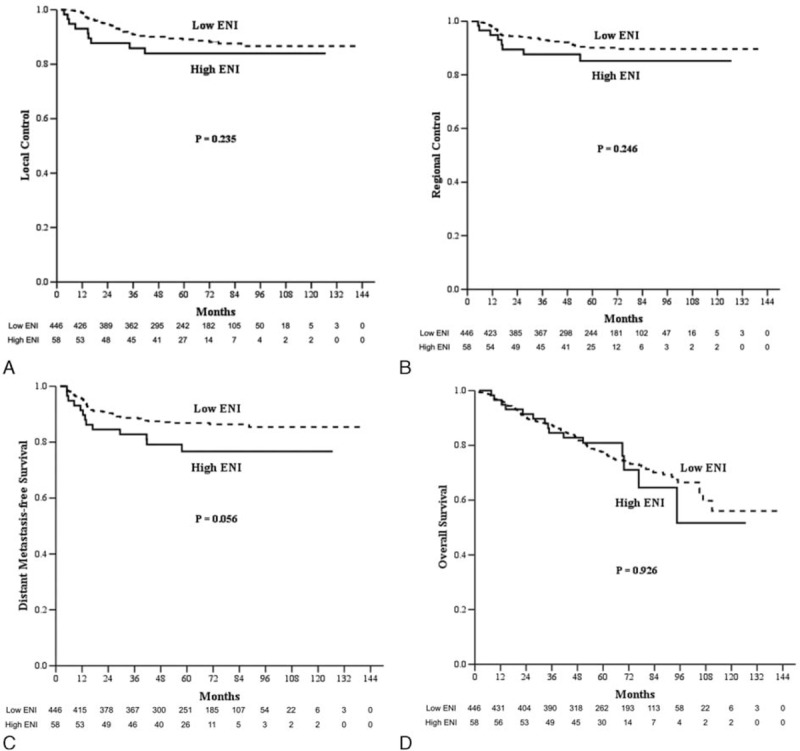
A, Local control. B, Regional control. C, Distant metastasis-free survival. D, Overall survival in the patients treated with low elective neck irradiation (low ENI, n = 446) and high elective neck irradiation (high ENI, n = 58) in all patients. ENI = elective neck irradiation.

**Figure 2:**
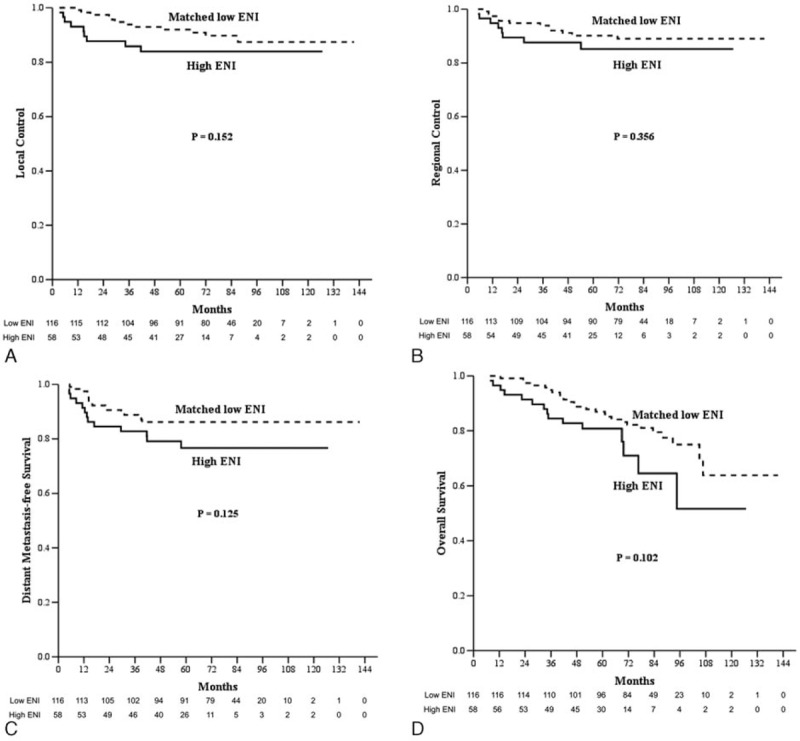
A, Local control. B, Regional control. C, Distant metastasis-free survival. D, Overall survival of patients in the matched low elective neck irradiation (matched low ENI, n = 116) and high elective neck irradiation (high ENI, n = 58) in the matched-pair analysis. CCRT = concurrent chemoradiotherapy, ENI = elective neck irradiation, IMRT = intensity-modulated radiotherapy, NPC = nasopharyngeal carcinoma, RT = radiation therapy.

The median follow-up for all patients was 63.5 months (range: 2.0–141.6 months), and all surviving patients underwent follow-up for at least 36 months. In all patients, the 5-year PFS, LC, RC, DMFS, OS, and CSS for low ENI and high ENI patients were 69.0% and 63.2% (*P* = 0.331), 89.0% and 83.9% (*P* = 0.235), 90.1% and 85.2% (*P* = 0.246), 86.8% and 76.6% (*P* = 0.056), 77.5% and 80.8% (*P* = 0.926), and 84.4% and 82.5% (*P* = 0.237), respectively (Fig. 1). In the multivariable analysis for all patients, all clinical variables (age, gender, pathology, T category, N category, chemotherapy, and the ENI group) were included in the Cox proportional hazard model, which revealed that the ENI group was not a significant factor for PFS, LC, RC, DMFS, OS, and CSS (Table 3). In the matched-pair analysis, the 5-year PFS, LC, RC, DMFS, OS, and CSS for matched low ENI and high ENI patients were 74.1% and 63.2% (*P* = 0.134), 92.0% and 83.9% (*P* = 0.152), 90.1% and 85.2% (*P* = 0.356), 86.2% and 76.6% (*P* = 0.125), 87.0% and 80.8% (*P* = 0.102), and 88.6% and 82.5% (*P* = 0.080), respectively (Fig. 2).

**Table 3:**
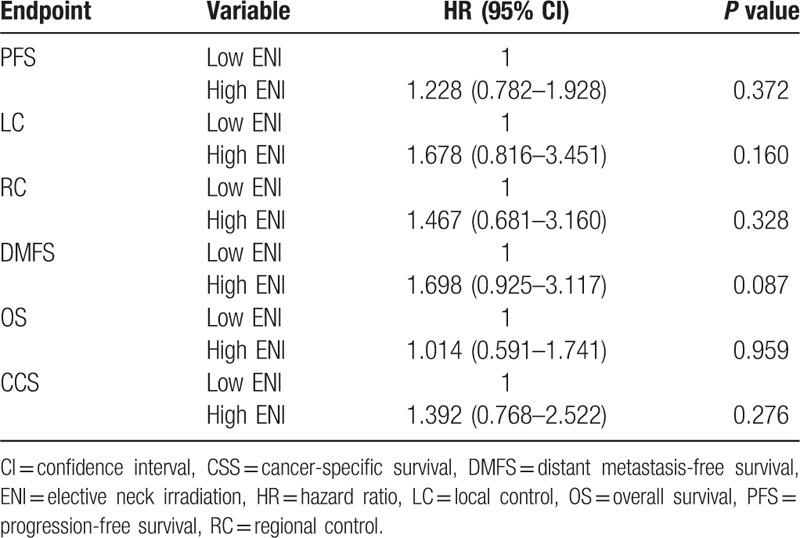
Multivariable analysis of all clinical variables in all patients (n = 504), including the ENI group.

At the end of the follow-up period, 127 patients had experienced disease relapse. Fifty-seven patients had a local recurrence, 48 patients failed regionally, and 71 patients developed distant metastasis. In the 48 patients with regional recurrence, 10 patients had a local disease failure followed by regional relapse, 13 patients had a regional recurrence in conjunction with a local disease failure, and 25 patients (25/504, 5.0%) had a regional recurrence as the first site of disease relapse. Among the 25 patients, 23 patients had neck failure in the initial gross nodal region, 20 were low ENI patients (20/446, 4.5%), and 3 in the high ENI group (3/58, 5.2%). No patients developed regional failure in the untreated level Ib region (the submandibular nodes). Two patients in the low ENI group (2/446, 0.45%) had a regional recurrence as the first site of disease relapse in the elective radiation field. One T4N1M0 male patient with initially right retropharyngeal gross node had an elective regional failure in the right level II lymph node, with 8 mm nodal size before the initial treatment with CCRT. He then received salvage surgery and was alive with disease 67.0 months after relapse. The other T1N3aM0 patient received RT alone as her initial treatment, had an initial 7 mm lymph node in the left supraclavicular fossa, experienced elective supraclavicular fossa regional failure with simultaneous distant metastasis. She received salvage chemotherapy and was dead due to disease 42.8 months after relapse. According to the initial treatment, the elective neck failure rate was 2.2% (1/46) for RT alone and 0.25% (1/400) for CCRT.

## 4 Discussion

Our results demonstrated that there was no significant difference in treatment outcome between the low and high ENI groups with long-term follow-up. The elective neck failure rate was only 0.45% in the low ENI group. The ENI dose of 46 Gy in 23 fractions may be sufficient to eradicate the microscopic disease of NPC, consistent with the suggestion by Gilbert H Fletcher in his traditional textbook.^[5]^ In addition, in prostate cancer, which is relatively radioresistant, the elective radiation dose for the pelvic nodes is also 46 to 50.4 Gy in conventional fractionation.^[3,4]^

The benefit of lower ENI is the reduced radiation dose to the pharynx and larynx, which may decrease the probability of RT-associated dysphagia and laryngeal dysfunction. According to the Quantitative Analyses of Normal Tissue Effects in the Clinic report, the risk of dysphagia rapidly increases from 45 to 60 Gy.^[10]^ A prospective randomized trial investigating a reduction of the ENI dose from 50 to 40 Gy in head and neck cancer revealed a significantly lower incidence of high-grade dysphagia in the low-dose arm without compromising disease control and survival.^[11]^ Chow et al^[12]^ reported that the cervical nodes from NPC are more radiosensitive to RT than the nodes from other head and neck cancers. The results of the prospective ENI dose deescalation trial for head and neck cancer^[11]^ may also be applicable to NPC.

The plasma Epstein–Barr virus (EBV) DNA^[13,14]^ has highly prognostic value in NPC, and the dose of ENI could be decided according to the EBV DNA titer. Xu et al^[15]^ showed that omitting chemotherapy in stage II NPC patients does not compromise the treatment outcome. So in patients with early stage and low plasma EBV DNA, avoid chemotherapy might be feasible to further reduce the toxicities of treatment.

The radiotherapeutic dose equivalent of concomitant chemotherapy in head and neck cancer is 7.2 to 12 Gy in 2 Gy per fraction.^[16,17]^ In our study, nearly 90% of the low ENI group patients had received CCRT, and the concomitant chemotherapy may have served as an RT enhancer, increasing control of the microscopic disease. In our study, the elective neck failure rate was 2.2% (1/46) in RT alone and 0.25% (1/400) in CCRT. CCRT for advanced NPC has become a routine practice after several prospective randomized trials established its solid level I evidence,^[18]^ and only a few patients with stage I disease would be treated with RT alone. The RT-enhancing effects of concomitant chemotherapy increase the safety of a low dose of ENI.

The involvement of submandibular nodes (level Ib) is rare in NPC, with an incidence of 2.2% to 3.1% in node-positive patients.^[8,19]^ In our study, the level Ib region was not included in the elective radiation field in the low ENI group, and no patient developed a regional failure in the untreated level Ib. In a prospective study of 323 NPC patients without elective irradiation of the level Ib region, Lin et al^[20]^ reported no regional recurrence in the submandibular nodes during a 3-year follow-up period. It has also been reported that the incidence of pathologically proven level Ib microscopic involvement is very low (approximately 3%) in early T-stage node-positive oropharyngeal cancer patients with negative finding on CT scans.^[21]^ Omitting RT to the submandibular nodes will better spare the oral cavity and submandibular glands, which may improve patient's salivary function and reduce patient-reported xerostomia.^[22]^ The importance of sparing radiation to the submandibular glands has also been demonstrated by a prospective Radiation Therapy Oncology Group trial.^[23]^ The importance of the submandibular glands in RT-induced xerostomia and health-related quality of life cannot be underestimated.

There are several limitations of the current study, including the retrospective nature of the study design and the inclusion of only patients who completed treatment, which could have affected the outcomes. Nevertheless, strength of this study is that it has a large number of NPC patients treated by IMRT and with long-term follow-up.

In conclusion, the present study shows that a low ENI dose of 46 Gy in 23 fractions without ENI to the submandibular nodes is feasible in NPC patients treated with IMRT, particularly in those undergoing concomitant chemotherapy. This idea needs to be further investigated in the future prospective studies.

## Acknowledgments

The authors thank for the help of statistical consultation (CLRPG3D0042) of the Biostatistical Center for Clinical Research (BCCR) in Chang Gung Memorial Hospital, Taoyuan, Taiwan. The authors also thank the members of the Head and Neck Oncology Team, Chang Gung Memorial Hospital and Chang Gung University, Taoyuan, Taiwan.
